# *Syzygium aromaticum* L. (Myrtaceae): Traditional Uses, Bioactive Chemical Constituents, Pharmacological and Toxicological Activities

**DOI:** 10.3390/biom10020202

**Published:** 2020-01-30

**Authors:** Gaber El-Saber Batiha, Luay M. Alkazmi, Lamiaa G. Wasef, Amany Magdy Beshbishy, Eman H. Nadwa, Eman K. Rashwan

**Affiliations:** 1 Department of Pharmacology and Therapeutics, Faculty of Veterinary Medicine, Damanhour University, Damanhour 22511, AlBeheira, Egypt; lamiaawasef@vetmed.dmu.edu.eg; 2 National Research Center for Protozoan Diseases, Obihiro University of Agriculture and Veterinary Medicine, Nishi 2-13, Inada-cho, Obihiro 080-8555, Hokkaido, Japan; amanimagdi2008@gmail.com; 3 Biology Department, Faculty of Applied Sciences, Umm Al-Qura University, Makkah 21955, Saudi Arabia; lmalkazmi@uqu.edu.sa; 4 Department of Pharmacology and Therapeutics, College of Medicine, Jouf University, Sakaka 72345, Saudi Arabia; emanhassannadwa@yahoo.co.uk; 5 Department of Medical Pharmacology, Faculty of Medicine, Cairo University, Giza 12613, Egypt; 6 Department of Physiology, College of Medicine, Al-Azhar University, Assuit 71524, Egypt; dremanrashwan2020@gmail.com; 7 Department of Physiology, College of Medicine, Jouf University, Sakaka 42421, Saudi Arabia

**Keywords:** *Syzygium aromaticum*, pharmacological activities, clove, essential oil, bioactive chemical constituents

## Abstract

Herbal medicinal products have been documented as a significant source for discovering new pharmaceutical molecules that have been used to treat serious diseases. Many plant species have been reported to have pharmacological activities attributable to their phytoconstituents such are glycosides, saponins, flavonoids, steroids, tannins, alkaloids, terpenes, etc. *Syzygium aromaticum* (clove) is a traditional spice that has been used for food preservation and possesses various pharmacological activities. *S. aromaticum* is rich in many phytochemicals as follows: sesquiterpenes, monoterpenes, hydrocarbon, and phenolic compounds. Eugenyl acetate, eugenol, and β-caryophyllene are the most significant phytochemicals in clove oil. Pharmacologically, *S. aromaticum* has been examined toward various pathogenic parasites and microorganisms, including pathogenic bacteria, *Plasmodium*, *Babesia*, *Theileria* parasites, *Herpes simplex*, and hepatitis C viruses. Several reports documented the analgesic, antioxidant, anticancer, antiseptic, anti-depressant, antispasmodic, anti-inflammatory, antiviral, antifungal, and antibacterial activity of eugenol against several pathogenic bacteria including methicillin-resistant *Staphylococcus epidermidis* and *S. aureus*. Moreover, eugenol was found to protect against CCl_4−_induced hepatotoxicity and showed a potential lethal efficacy against the multiplication of various parasites including *Giardia lamblia*, *Fasciola gigantica*, *Haemonchus contortus*, and *Schistosoma mansoni*. This review examines the phytochemical composition and biological activities of clove extracts along with clove essential oil and the main active compound, eugenol, and implicates new findings from gas chromatography-mass spectroscopy (GC-MS) analysis.

## 1. Introduction

The traditional medicinal system based on the use of herbal remedies still plays an important role in the health care system. In recent decades, medicinal plants have been gaining wider acceptance due to the perception that these plants being natural products have lesser side effects and improved efficacy than their synthetic counterparts [1,2]. Currently, about 80% of the world’s inhabitants rely on traditional medicines as a major form of their primary health care [3]. Pharmacologically, various herbal plants possess bactericidal, virucidal, fungicidal activities; they are used in embalmment, in food preservation, and have anti-inflammatory, antimicrobial, spasmolytic, sedative, analgesic, and local anesthetic activities [4,5]. Many plant species have been reported to have pharmacological activities attributable to their phytoconstituents such are glycosides, saponins, flavonoids, steroids, tannins, alkaloids, terpenes and accordingly [4]. Up to date, herbal remedies have been documented as a vital source for discovering novel pharmaceutical molecules that have been used to treat serious diseases. These identified phytochemicals have been considered a remarkable leading compound in the search for effective and new drugs [5].

*Syzygium* (*S.*) *aromaticum*, also known as clove, is a dried flower bud belonging to the Myrtaceae family that is indigenous to the Maluku islands in Indonesia but has recently been farmed in different places worldwide [6,7]. The clove tree is composed of leaves and buds (the commercial part of the tree) and the flowering bud production begins four years after plantation. Afterward, they are collected either by hand or using a natural phytohormone in the pre-flowering stage [6]. Interestingly, they are commercially used for many medicinal purposes and in the perfume industry, and clove is considered one of the spices that can be potentially used as preservatives in many foods, especially in meat processing, to replace chemical preservatives due to their antioxidant and antimicrobial properties [6,8]. Several reports have documented the antibacterial, antiviral, anticarcinogenic, and antifungal activities of some aromatic herbs including cinnamon, oregano, clove, thyme, and mint. However, clove has gained much attention among other spices due to its potent antimicrobial and antioxidant activities [9]. The effective role of clove in the inhibition of different degenerative diseases is attributed to the presence of various chemical constituents in high concentrations with antioxidant activity [10,11]. Clove essential oil (CEO) is traditionally used in the treatment of burns and wounds, and as a pain reliever in dental care as well as treating tooth infections and toothache. In addition to that, its use has been documented in various industrial applications and is used extensively in perfumes, soaps and as a cleansing vehicle in histological work [12]. Cloves are used in Indian and Chinese traditional medicine as a warming and stimulating agent [7]. Traditionally, cloves have been used for centuries in the treatment of vomiting; flatulence; nausea; liver, bowel and stomach disorders; and as a stimulant for the nerves. In tropical Asia, cloves have been documented to relieve different microorganisms as scabies, cholera, malaria, and tuberculosis. As well, in America, clove has been traditionally used in inhibiting food-borne pathogens to treat viruses, worms, candida, and different bacterial and protozoan infections [13]. Moreover, eugenol has been widely used in dentistry because it can penetrate the dental pulp tissue and enter the bloodstream [14]. Sesquiterpenes, isolated from clove were reported to have anti-carcinogenic activity [15].

## 2. Chemical Constituents

Pharmacologically, clove has been documented as the main source of phenolic molecules like hidroxibenzoic acids, flavonoids, hidroxiphenyl propens, hidroxicinamic acids, and eugenol (C_10_H_12_O_2_)—which is the major bioactive molecule—and gallic acid derivatives like hidrolizable tannins that are found in high amounts in the fresh plant (Table 1) [6,9,16]. Moreover, clove contains flavonoids namely quercetin and kaempferol and phenolic acids like ferulic, caffeic, ellagic, and salicylic acids [6]. Clove flower buds contain up to 18% of essential oil which consists of eugenol, eugenol acetate and β-cariofileno [17]. Clove oil is colorless or pale yellow with a distinct clove flavor and taste. The differences in CEO content and composition depend mainly on several factors like pre-treatments, variety, agro-ecological conditions, and extraction processes [18]. Notably, Gülçin [19] reported the in vitro antioxidant effectiveness of eugenol and discussed the relationship between structure and activity. They showed that eugenol allows the donation of the hydrogen atom and subsequently fixes the phenoxil radical, which results in the formation of steady molecules that do not establish or increase oxidation. Additionally, the eugenol compound has a pleasant carbon chain link with the aromatic ring which can be involved in phenoxil radical stabilization by resonance. Gas chromatography-mass spectroscopy (GC-MS) analysis demonstrated the existence of 36 components in the CEO that was isolated by hydro-distillation including eugenol, β-caryophyllene, eugenylacetate, ethyl hexanoate, 2-heptanone, α-humulene, calacorene, humulenol, and calamen-ene [20,21,22,23].

## 3. Crude Clove Extracts Efficacies

Several *S. aromaticum* molecules namely kaempferol, biflorin, 5, 7-dihydroxy-2-methylchromone-8-C-β-D-glucopyranoside, orsellinic acid glucoside, myricetin, rhamnocitrin, gallic acid, oleanolic acid, ellagic acid, and flavonoids triglycosides have been documented for their effectiveness in inhibiting oral pathogens [24]. As the ethanolic *S. aromaticum* extract showed high antioxidant efficacy in addition to its hepatoprotective activity on liver damage caused by paracetamol treatment [25]. The possible explanation of increased serum enzymes in paracetamol-induced liver damage may be attributed to inhibition of intracellular enzymes through membrane stabilization efficacy, which corresponds to the view that the serum transaminases levels have been restored by recovering hepatic *Pseudomonas aeruginosa* and *Escherichia coli* renchyma and the hepatocytes regeneration [26]. Essawi and Srour [27] tested the antimicrobial efficacy of six medicinal herbal extracts in vitro toward four bacterial species methicillin-resistant *Staphylococcus aureus* and *Bacillus subtilis* were the most inhibited microorganisms. *Syzygium aromaticum* extract was the most active against multidrug-resistant. Joshi et al. [28] found that *S. aromaticum* was the most effective against *Salmonella typhi*. Moreover, Jirovetz et al. [17] showed that the flower bud extract of *S. aromaticum* (clove) showed antibacterial efficacy toward *Bacillus* and *Serratia marcescens* bacterial isolates. In addition, Oulkheir et al. [29] found that the CEO produced an inhibition zone against *E. coli* of 16 mm and a higher inhibitory zone (20 mm) against *Salmonella* species, while no antibacterial effect on *K. pneumoniae*. Haroun and Al-Kayali [30] noticed good synergism between ethanolic extract from *S. aromaticum* with different antibiotics compared with water extract against *S. aureus* isolate. Interestingly, previous reports investigated the antifungal effectiveness of eugenol and clove oil against yeasts, filamentous, and human pathogenic fungi [31,32,33,34]. Moreover, Nejad et al. [35] reported the antibacterial efficacy of various natural bioactive molecules namely thymol, eugenol, carvacrol, and cinnamaldehyde against the *E. coli*, and they revealed that eugenol resulted in the lowest antibacterial efficacy, whilst carvacrol and thymol, cinnamaldehyde and eugenol combined treatment revealed synergistic efficacy [36].

## 4. Biological Activities

### 4.1. Biological/Biochemical Properties of S. aromaticum

**Figure 1:**
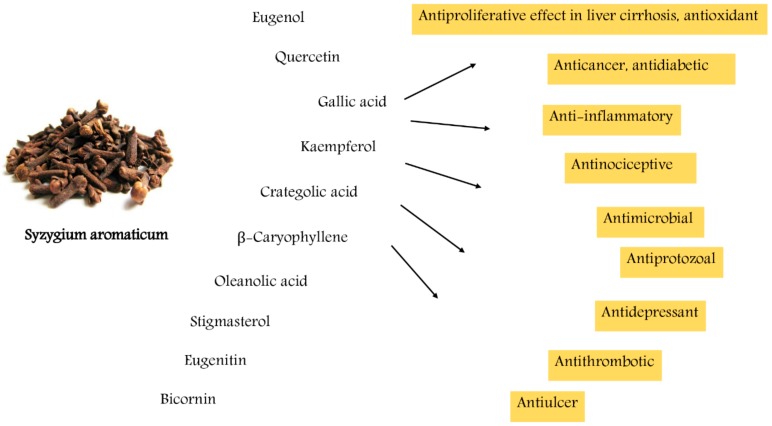
The biological activities of *S. aromaticum* and its related compounds.

Han and Parker [37] have revealed the antiviral, antimicrobial, antifungal, anticancer, antioxidant, and anti-inflammatory activities of the CEO and its main active constituent eugenol, and they revealed that CEO influenced the cancer biology and cell cycle control. The problems of pathogen resistance, as well as the toxic residues to most of the commercially available antimicrobial drugs severely weaken their effective curative and protective approaches [37,38,39]. Therefore, it is clear that the development of new and effective antimicrobial treatment options is vital for improving disease treatment and control. Clove is a well-known and significant herbal remedy because of its broad pharmacological efficacy [40]. Recent studies have examined the in vivo increase in the lipid peroxidation and blood sugar in diabetic rats and reestablished the levels of the antioxidant enzyme after nutrition supplement with cloves [41]. Additionally, Shukri et al. [42] revealed that the dietary cloves in vivo reduced the tissue damages in the livers, lens, and cardiac muscles in rats. Pharmacologically, clove oil is used in a wide range as an antiseptic in oral diseases and for the treatment of toothaches, allergy disorders, asthma, acne, scars, and rheumatoid arthritis, and it showed antispasmodic and acaricidal effects toward *Dermatophagoides pteronyssinus* and *Dermatophagoides farina* [43,44]. Moreover, the CEO has shown aphrodisiac, antipyretic, appetizer, hypnotic, anxiolytic, antiemetic, analgesic, decongestant, antimicrobial, antiepileptic, myorelaxant, anti-inflammatory, and expectorant properties as well as has a medicinal influence against trophic disorder [37,45]. Notably, tannins, ellagic acid, gallic acid, flavonoids and their glycosides isolated from alcoholic and aqueous clove buds extracts were reported to have antithrombotic, antiprotozoal, hypoglycemic, anti-inflammatory, gastroprotective, and aphrodisiac efficacy [46,47,48,49,50]. In traditional medicine, clove has been used in flatulence, indigestion complaints and diarrhea [51]. The biological activities of *S. aromaticum* and its related compounds are shown in Figure 1.

Several reports demonstrated the antimicrobial efficacies of clove against different fungal and bacterial strains. For instance, Sofia et al. [52] examined the antimicrobial efficacy of several Indian spice herbs (e.g., ginger, garlic, mint, clove mustard, and cinnamon). Moreover, Dorman and Deans [53] evaluated the antibacterial efficacy of thyme, clove, geranium, nutmeg, oregano, and black pepper toward 25 strains of gram-negative and gram-positive bacteria. Thielmann et al. [54] documented that thyme, bay, oregano, and CEOs demonstrated different grades of inhibition against *E. coli*. As well, carvacrol and eugenol enclosed in a non-ionic surfactant were examined toward *Listeria monocitogenes* and *E. coli*, and the results revealed the effectiveness of eugenol to suppress these microorganisms multiplication [55]. Rana et al. [56] documented the antifungal efficacy of clove oil towards *Trichophyton rubrum*, *Microsporum canis*, *T. mentagrophytes*, *Fusarium monoliforme*, *M. gypseum*, *F. oxysporum, Epidermophyton floccosum*, *Mucor* sp., *M. gypseum*, *T. rubrum*, and *Aspergillus* sp. [57]. Fu et al. [58] as well as Palombo and Semple [59] reported the antibacterial effect of pure clove oil either alone or combined with rosemary oil towards *P. aeruginosa*, *B. subtilis*, *S. epidermidis*, *S. aureus*, *Proteus vulgaris*, *E. coli*, and methicillin-resistant *S. epidermidis* and *S. aureus*. Additionally, herbal remedies have been documented as a major source for discovering new pharmaceutical molecules to inhibit and control viral infections [60]. Eugeniin, the compound isolated from *S. aromaticum* extract has been documented for its antiviral efficacy towards various herpes virus strains and the hepatitis C virus by its action on the synthesis of the viral DNA by inhibiting the viral DNA polymerase enzyme [6,61]. Another research revealed the antiviral efficacy of *S. aromaticum* aqueous extracts against herpes simplex virus type 1 (HSV-1) and influenza A virus when combined with acyclovir [62,63,64,65]. The possible antimicrobial action for clove oil is attributed to eugenol which consists of about 85% to 92% of total clove oil content [66].

Carvacrol and eugenol are the main components of clove responsible for its fungicidal characteristics against onychomycosis isolated fungi, *T. mentagrophytes* and *Candida albicans* [31,67,68]. Interestingly, Núñez et al. [69] reported the fungicidal activity of the mixture of a concentrated sugar solution with clove oleoresin by decreasing the inoculum size of fungi. Chami et al. [70] revealed remarkable morphological deterioration with cellular deformation in *Saccharomyces cerevisiae* cells caused by clove oil. Siripornvisal et al. [71] as well as Pinto et al. [72], examined the potent inhibitory antifungal effects of the CEO towards the mycelial multiplication of *Botrytis cinerea* and collection strains of dermatophyte, *Aspergillus* and *Candida* species. Recently, Batiha et al. [7] documented the antipiroplasmic effect of *S. aromaticum* methanolic extracts against piroplasm parasites multiplication. Moreover, the previous study described the in vitro antiplasmodial efficacy of methanolic *S. aromaticum* extracts against a chloroquine-resistant strain of *Plasmodium falciparum*, the closely related apicomplexan parasite to *Babesia* and *Theileria* [73].

### 4.2. Efficacy in Diseases

Several in vitro methods like 1, 1-diphenyl-2-picryl hydroxyl (DPPH) radical, b-carotene-linoleate, ferric thiocyanate, and hydroxyl radical revealed that caraway and clove antioxidant activity is consistent with the synthetic food preservative, butylated hydroxytoluene (BHT) [74]. Moreover, Gülçin et al. [75] measured the scavenging of the DPPH radical of clove oil in comparison to some artificial antioxidant agents, namely, alfa-tocopherol, BHT, Trolox, and butylated hydroxyanisole, and they demonstrated that the clove oil antioxidant activity declined as follows: clove oil > BHT > alfa-tocopherol > butylated hydroxyanisole > Trolox. Various in vitro methods including DPPH, oxygen radical absorbance capacity, ferric reducing antioxidant power, 2-deoxiguanosine, 2, 2′-azino-bis (3-ethylbenzothiazoline-6-sulphonic acid) (ABTS), and xanthine oxidase used to examine the antioxidant activity of aqueous *S. aromaticum* extract. They documented that the potent antioxidant efficacy of aqueous *S. aromaticum* extract may be due to the strong hydrogen donating ability, scavenging of hydrogen peroxide, free radicals and superoxide and metal chelating ability [76]. Antioxidant agents like clove extracts and the CEO play a significant role in treating memory deficits resulting from oxidative stress [77]. Halder et al. [78] revealed that CEO’s pretreatment reduced the oxidative stress evaluated by glutathione as well as malondialdehyde levels in mice’s brains. They concluded that the ability of clove oil to restore memory and learning deficiencies resulted from short- and long-term scopolamine treatment is attributed to its effectiveness in reducing oxidative stress. 

Moreover, the analgesic effect of clove, as well as eugenol, have been documented against toothache, joint pain by activating chloride and calcium channels in ganglionar cells [79]. However, another study revealed that the analgesic activity of clove may be attributed to its capsaicin agonist activity [80]. Daniel et al. [81] reported the in vivo analgesic efficacy of eugenol using the abdominal wriggling method stimulated by acetic acid. Interestingly, the anti-carcinogenic and cytotoxic activities of the CEO have been reported against human tumor cell lines PC-3 and Hep G2 [15,82,83]. Chaieb et al. [23] documented that eugenol and dehydrodieugenol have been shown to stimulate human cancer cell death. Moreover, the antimutagenic efficacy of cinnamaldehyde has been investigated against human-derived hepatoma cells, as it inhibited the micronuclei incidence caused by different heterocyclic amines [84]. Natural products have confirmed to be the most efficient in terms of their ability to change the function of proteins related to cancer [85]. Kouidhi et al. [86] and Kumar et al. [87] established that CEO and eugenol possess anticancer activities against leukemia, lung, breast, and colorectal cancer cells. Clove exerted anti-inflammatory and immunomodulatory activities by suppressing the lipopolysaccharide (LPS) action as well as the nuclear factor-κB (NF-κB) pathway. Han and Parker [37] reported that the anti-inflammatory activity of clove may be related to the active compound, eugenol.

Moreover, eugenol was found to protect against hepatotoxicity caused by CCl_4_ when administered with CCl_4_ therapy [88]. Interestingly, other phytochemical compounds isolated from *S. aromaticum* extracts including sanguinarine and benzo phenanthridine alkaloids have been documented for protection from liver damage [89]. Shyamala et al. [90] proved that clove intake tends to recover ALT, urea, AST, and lipid levels in kidneys, serum, and liver in comparison with normal values in hyperlipidemic rats. The antidiabetic efficacy of *S. aromaticum* extracts may be attributed to the existence of insulin-stimulating agents [91]. In vivo experiments revealed that the normal blood sugar has been enhanced in *S. aromaticum* extracts-treated mice [91,92]. It was found that the CEO contains many biologically active compounds with potent gastroprotective activities and this activity has been found due to its high flavonoid contents [93]. The in vitro and in vivo experiments have documented the antiobesity efficacy of *S. aromaticum* extracts by reducing the serum triglycerides and cholesterol levels [94]. Additionally, Jung et al. [95] documented that diet supplemented with *S. aromaticum* extracts decreased serum insulin, leptin, and hepatic lipid levels along with the body weight of high-fat diet mice, suggesting its prospect as a natural anti-obesity supplement and its ability to decrease the hepatic lipid accumulation.

Clove essential oils have been reported to increase blood circulation and raise body temperature [6]. Several reports documented that clove can reduce the risk of arterial sclerosis, cardiovascular disorders, and other disease associated with oxidative stress. Eugenol also exhibits reversible, dose-related vasodilator as well as negative inotropic activities in heart muscle and showed smooth muscle relaxant and hypotensive efficacy [96]. Clove has been documented to possess nervous stimulating as well as sexual behavior boosting effect in male mice [97], and this action may be attributed to their nervine enhancing activity. Moreover, it showed an increase in mating performance in mice compared to an increase in sexual motivation [97]. Cortés-Rojas et al. [6] reported the ability of clove oil to inhibit prevent premature ejaculation. The sexual behavior of clove in humans has been enhanced by stimulating the testosterone level. Clove oil has been documented as thromboxane synthesis and platelet aggregation inhibitors and showed an anticoagulant activity. Moreover, clove oil prevented the platelet aggregation caused by the platelet-activating factor, arachidonic acid or collagen, and the results revealed that clove oil is more efficient in inhibiting platelet-activating factor- and arachidonic acid-induced aggregation than collagen [98]. As well, eugenol was reported to prevent prostaglandin biosynthesis, thromboxane B2 formation, and platelet aggregation caused by arachidonic acid in vitro [99]. The myogenic antispasmodic effect of eugenol has been documented on the airway smooth muscle of rats. It was found to act by blocking Ca^2+^ channels managed by voltage and receptors, enhancing the release of Ca^2+^ from the sarcoplasmic reticulum and decreasing the sensitivity of the contractile proteins to Ca^2+^ [100]. In addition to that, it showed an antipyretic effect through a central action comparable with that of acetaminophen and allopathic antipyretic agents [101]. Eugenol and its analogs revealed anti-depressant efficacy in vivo by preventing monoamine oxidase [102].

### 4.3. Efficacy of the Most Common Compound Eugenol

Clove essential oil and eugenol derived from *S. aromaticum* have been documented to possess useful analgesic, anesthetic, and antiseptic effects and are therefore commonly used in dentistry [23]. In addition to that, they showed an anti-inflammatory efficacy against murine macrophages by suppressing the pro-inflammatory cytokines production [103,104] and eugenol prohibited IL-8 production enhancement against human gingival fibroblasts (HGF) but not against skin keratinocytes (HaCat) or periodontal ligament fibroblasts (HPLF) [41]. Eugenol showed a strong antibacterial efficacy against different strains of Gram-positive and Gram-negative bacteria and revealed greater antimicrobial activity when combined with gentamicin, β-lactam and vancomycin antibiotics [58,59]. The antifungal efficacy of eugenol and clove oil has been investigated towards yeasts and filamentous fungi, including various human pathogenic fungi and food-borne fungal species [23,31,32,33]. Interestingly, several reports documented that eugenol isolated from *S. aromaticum* extracts have shown potent trypanocidal as well as leishmanicidal efficacy against *Trypanosoma cruzi*, *Leishmania donovani*, *L. amazonensis*, *L. major* and *L. tropica* [105,106]. Additionally, eugenol showed a potential lethal efficacy against the growth and multiplication of various parasites including *Giardia lamblia*, *Fasciola gigantica*, *Haemonchus contortus*, and *Schistosoma mansoni* [107,108]. Eugenol exhibited antiviral activity against HSV-1 and herpes simplex -2 (HSV-2) by preventing viral replication and reducing the viral infection [64]. Eugenol isolated from *S. aromaticum* extracts and their essential oils has shown its free radical scavenging, antioxidant, and antimicrobial properties [58,109].

The anti-inflammatory effects of eugenol were attributed to its effect to prevent neutrophil/macrophage chemotaxis and prostaglandin synthesis as well as cyclooxygenase II enzyme expressions [35]. Moreover, eugenol dimers exhibited a chemopreventive effect by inhibiting the cytokines expression in macrophages [110]. Eugenol has been suggested to possess recovery effects on arthritis and thus can be used in the treatment of arthritis [111]. Kim et al. [112] investigated the direct effect of eugenol in inhibiting NF-κB activation caused by tumor necrosis factor (TNFα) and preventing cyclooxygenase activity (COX-2) in LPS stimulated macrophages with IC_50_ value equal to 2.7 μM in the healthy cells. Additionally, eugenol protected macrophages cellular dysfunction caused by chemicals and stabilized the pro/anti-inflammatory mediators. Eugenol has been investigated for its anti-cancer activity against skin tumors, melanoma, gastric cancer, leukemia, and prostate cancer by oncogene regulation and the caspase-dependent pathway. For instance, eugenol and biphenyl (S)-6, 6′-dibromo-dehydrodieugenol provokes antiproliferative efficacy on neuroectodermal tumor cells by stimulating partial apoptosis [113]. The epoxide form of eugenol has been reported as a potent therapy for stimulating apoptosis in human breast cancer cells [114]. Moreover, eugenol prevented various oncogenes-related breast cancer namely, NF-κB and cyclin D1 as well as blocked the breast cancer multiplication in a p53- independent manner and upregulated the flexible cyclin-dependent kinase inhibitor protein and this anti-proliferative activity was significantly noticed in xenograft human breast tumors. In vitro and in vivo studies demonstrated the anti-breast cancer activities of eugenol, suggesting that it could be used to enhance breast cancer treatment by targeting the E2F1/survivin pathway. Eugenol cytotoxic concentrations caused the ATP reduction and enhancing the glycolytic metabolites and polyamines in normal oral cells and oral squamous cell carcinoma, indicating the unprogrammed cell death induction [114]. Nam and Kim [115] revealed the ability of eugenol in preventing metastasis associated with oxidative stress by blocking the efficacy of matrix metalloproteinase-9 in PMA-induced HT1080 cells. Nowadays, combination chemotherapies are being reported as the most significant strategy for alleviating serious diseases, including cancer, to decrease the dose of the drugs, leading to a reduction in their toxic symptoms as well as drug resistance. Interestingly, Hemaiswarya and Doble [116] showed that eugenol and 5-fluorouracil combined treatment displayed a more cytotoxic effect toward cervical cancer cells (HeLa) indicating that eugenol is a good combinatorial agent by inducing cancer cells apoptosis.

Eugenol and clove oil have been documented to have a potent effect on fatty liver and dyslipidemia by a different mechanism of action [117], which involves oxidative stress by reducing the oxidative damage [118]. In vivo experiments showed that eugenol administration at 100 mg/kg 4 days prior to and 6 days together with gentamicin suppressing the oxidative damage caused by gentamicin [119]. The antiulcer efficacy of eugenol may be associated with the presence of several factors that increase gastric mucus production and barrier resistance [120]. Moreover, Oliveira et al. [120] documented that eugenol pretreatment in rats decreased the gastric acid secretion, gastric ulcers, and pepsin activity caused by indomethacin treatment and increased the gastric mucin concentration.

## 5. Pharmacokinetics Studies of Eugenol

Eugenol metabolism has been examined in male and female healthy volunteers. Eugenol is known to be easily absorbed after oral administration and rapidly reaches plasma and blood with a half-life of 14 and 18 h, respectively and its accumulative impact has been observed after its daily administration for treating neuropathic pain [101]. Afterward, eugenol is metabolized to glucuronic acid or sulfate conjugate in the liver. Methyleugenol was partially metabolized in the liver by the action of different CYP 450 enzymes to reactive 2′, 3′-(allylic) epoxide or 1′ hydroxy-derivatives [121]. Eugenol metabolism has been indicated by the same bioactivation pathway and the genotoxic and carcinogenic form of eugenol seems likely to be insignificant when compared to methyleugenol and is excreted in the conjugated form in the urine during 24 h [122]. Secondary metabolic pathways include the oxidation of side-chain double bond to the epoxide and then hydrolyzation to diol and additional oxidation to isoeugenol accompanied by allylic oxidation and then reducing the side-chain double bond [122]. Less than 0.1% of the eugenol dose was secreted in the unmetabolized form in the urine, while 95% of its dose was restored in the urine, greater than 99% composed of phenolic conjugates and 50% were found as eugenol-glucuronide and sulphate. The urine consists of eugenol conjugates and other metabolites (e.g., cis- and trans-isoeugenol, 4-hydroxy-3-methoxyphenyl-propane, 3-(4- hydroxy-3-methoxyphenyl)-propionic acid, 3-(4-hydroxy-3-methoxyphenyl)-propane-1, 2-diol, and 3-(4-hydroxy3-methoxyphenyl)-propylene-1, 2-oxide) [121].

## 6. Toxicity Doses

Food and Drug Administration (FDA) has confirmed the safety of clove buds, clove oil, eugenol, and oleoresins as a food supplement; however, there has been considerable attention regarding its toxicity recently [123]. Prashar et al. [21] have examined the cytotoxic activities of CEO and eugenol in vitro against human fibroblasts and endothelial cells, and they documented they recognized them as safe. On the other hand, other reports revealed that eugenol has an allergic efficacy when used in dentistry [12,124]. Moreover, eugenol, as well as CEO, was reported to have a spermicidal effect in vitro in six male partners of infertile couples [125]. The World Health Organization (WHO) has proven that the acceptable daily amount of clove in humans is 2.5 mg/kg body weight [82]. The CEO toxic activity was assessed in *Poecilia reticulata* and *Danio rerio* aquarium fish species and it exhibited half-maximal lethal concentrations (LD_50_) at 18.2 ± 5.52 and 21.7 ± 0.8 mg/mL against *Danio rerio* and *Poecilia reticulata* after 96 h, respectively [126]. Janes et al. [127] documented the acute side effects (e.g., disseminated intravascular coagulopathy, generalized seizures, and hepatotoxicity after CEO administration). Recently, Johannah et al. [50] demonstrated the remarkable detoxification and the cardiac health effects in humans by reducing lipid peroxidation and increasing the endogenous redox enzyme levels. Moreover, another in vivo study reported the allergic contact dermatitis of eugenol in guinea pigs [75].

## 7. Conclusions

This review examines the medicinal properties and all phytochemical molecules isolated from *S. aromaticum*. Carvacrol, eugenol, thymol, and cinnamaldehyde are the major constituents extracted from the CEO. Eugenol is the active substance of the CEO, and the FDA considered it a safe substance in general. The daily allowable human consumption of clove oil approved by the WHO Expert Committee on Food additives is 2.5 mg/kg body weight. Pharmacologically, clove and its main constituents possess antimicrobial, antioxidant, anti-inflammatory, analgesic, anticancer, and anesthetic effects. Moreover, they showed insecticidal, mosquito repellant, aphrodisiac, and antipyretic activities.

## Figures and Tables

**Table 1:** International Union of Pure and Applied Chemistry (IUPAC) name and chemical formula of bioactive molecules isolated from *S. aromaticum*.

Compd.	IUPAC Name	Chemical Formula	Compd.	IUPAC Name	Chemical Formula
**Eugenol**	2-Methoxy-4-(prop-2-en-1-yl)phenol	C_10_H_12_O_2_	**Gallic acid**	3,4,5-Trihydroxybenzoic acid	C_7_H_6_O_5_
**β-Caryophyllene**	(1*R*,4*E*,9*S*)-4,11,11-Trimethyl-8-methylidenebicyclo[7.2.0]undec-4-ene	C_15_H_24_	**Biflorin**	5,7-dihydroxy-2-methyl-6-[(2*S*,3*R*,4*R*,5*S*,6*R*)-3,4,5-trihydroxy-6-(hydroxymethyl)oxan-2-yl]chromen-4-one	C_16_H_18_O_9_
**Vanillin**	4-Hydroxy-3-methoxybenzaldehyde	C_8_H_8_O_3_	**Myricetin**	3,5,7-Trihydroxy-2-(3,4,5-trihydroxyphenyl)-4-chromenone	C_15_H_10_O_8_
**Crategolic acid (Maslinic acid)**	(4a*S*,6a*R*,6a*S*,6b*R*,8a*R*,10*R*,11*R*,12a*R*,14b*S*)-10,11-dihydroxy-2,2,6a,6b,9,9,12a-heptamethyl-1,3,4,5,6,6a,7,8,8a,10,11,12,13,14b-tetradecahydropicene-4a-carboxylic acid	C_30_H_48_O_4_	**Campesterol**	(3*S*,8*S*,9*S*,10*R*,13*R*,14*S*,17*R*)-17-[(2*R*,5*R*)-5,6-dimethylheptan-2-yl]-10,13-dimethyl-2,3,4,7,8,9,11,12,14,15,16,17-dodecahydro-1*H*-cyclopenta[*a*]phenanthren-3-ol	C_28_H_48_O
**Kaempferol**	3,5,7-Trihydroxy-2-(4-hydroxyphenyl)-4*H*-chromen-4-one	C_15_H_10_O_6_	**Stigmasterol**	3*S*,8*S*,9*S*,10*R*,13*R*,14*S*,17*R*)-17-[(*E*,2*R*,5*S*)-5-ethyl-6-methylhept-3-en-2-yl]-10,13-dimethyl-2,3,4,7,8,9,11,12,14,15,16,17-dodecahydro-1*H*-cyclopenta[*a*]phenanthren-3-ol	C_29_H_48_O
**Rhamnetin**	2-(3,4-dihydroxyphenyl)-3,5-dihydroxy-7-methoxychromen-4-one	C_16_H_12_O_7_	**Oleanolic acid**	(4a*S*,6a*R*,6a*S*,6b*R*,8a*R*,10*S*,12a*R*,14b*S*)-10-hydroxy-2,2,6a,6b,9,9,12a-heptamethyl-1,3,4,5,6,6a,7,8,8a,10,11,12,13,14b-tetradecahydropicene-4a-carboxylic acid	C_30_H_48_O_3_
**Eugenitin**	5-Hydroxy-7-methoxy-2,6-dimethylchromen-4-one	C_12_H_12_O_4_	**Bicornin**	[(12R,14S,15R,16R,17R)-4,5,6,22,23,29,30-heptahydroxy-9,19,26-trioxo-14,15-bis[(3,4,5-trihydroxybenzoyl)oxy]-2,10,13,18,25-pentaoxahexacyclo[18.9.3.03,8.012,17.024,32.027,31]dotriaconta-1(29),3,5,7,20,22,24(32),27,30-nonaen-16-yl] 3,4,5-trihydroxybenzoate	C_48_H_32_O_30_
**Eugenin**	5-Hydroxy-7-methoxy-2-methylchromen-4-one	C_11_H_10_O_4_	**Quercetin**	2-(3,4-dihydroxyphenyl)-3,5,7-trihydroxychromen-4-one	C_15_H_10_O_7_
**Ellagic acid**	2,3,7,8-Tetrahydroxy-chromeno[5,4,3-cde]chromene-5,10-dione	C_14_H_6_O_8_	**Carvacrol**	2-methyl-5-propan-2-ylphenol	C_10_H_14_O

## Abbreviations

CCl_4_: carbon tetrachloride; GC-MS; Gas chromatography-mass spectroscopy; *S. aromaticum*: *Syzygium aromaticum*; IUPAC: International Union of Pure and Applied Chemistry; CEO: clove essential oil; HSV-1: Herpes simplex virus type 1; HSV-2: herpes simplex -2; DPPH: 1, 1-diphenyl-2-picryl hydroxyl; BHT: butylated hydroxytoluene; ABTS: 2, 2′-azino-bis (3-ethylbenzothiazoline-6-sulphonic acid); LPS: lipopolysaccharide; HGF: human gingival fibroblasts; HaCat: skin keratinocytes; HPLF: periodontal ligament fibroblasts; NF-κB: nuclear factor-κB; TNFα: tumor necrosis factor; COX-2: cyclooxygenase activity; FDA: Food and Drug Administration; WHO: World Health Organization; LD_50_: half-maximal lethal concentrations.
